# Investigating patient eligibility for anti-amyloid monoclonal antibody treatment of Alzheimer's disease: real-world data from an Austrian psychiatric memory clinic population

**DOI:** 10.1192/bjo.2024.747

**Published:** 2024-09-23

**Authors:** Michaela Defrancesco, Elke R. Gizewski, Stephanie Mangesius, Malik Galijasevic, Irene Virgolini, Alexander Kroiss, Josef Marksteiner, Juliane Jehle, Burak Doganyigit, Alex Hofer

**Affiliations:** University Clinic for Psychiatry I, Department of Psychiatry, Psychotherapy, Psychosomatics and Medical Psychology, Medical University of Innsbruck, Austria; Department of Radiology, Medical University of Innsbruck, Austria; and Neuroimaging Core Facility, Medical University of Innsbruck, Austria; Department of Nuclear Medicine, Medical University of Innsbruck, Austria; Department of Psychiatry and Psychotherapy A, State Hospital of Hall in Tirol, Austria

**Keywords:** Alzheimer's disease, mild cognitive impairment, lecanemab, anti-amyloid therapy, memory clinic

## Abstract

**Background:**

Pharmacological treatment options for patients with dementia owing to Alzheimer's disease are limited to symptomatic therapy. Recently, the US Food and Drug Administration approved the monoclonal antibody lecanemab for the treatment of amyloid-positive patients with mild cognitive impairment (MCI) and early Alzheimer´s dementia. European approval is expected in 2024. Data on the applicability and eligibility for treatment with anti-amyloid monoclonal antibodies outside of a study population are lacking.

**Aims:**

This study examined eligibility criteria for lecanemab in a real-world memory clinic population between 1 January 2022 and 31 July 2023.

**Method:**

We conducted a retrospective, single-centre study applying the clinical trial eligibility criteria for lecanemab to out-patients of a specialised psychiatric memory clinic. Eligibility for anti-amyloid treatment was assessed following the phase 3 inclusion and exclusion criteria and the published recommendations for lecanemab.

**Results:**

The study population consisted of 587 out-patients. Two-thirds were diagnosed with Alzheimer's disease (probable or possible Alzheimer's disease dementia in 43.6% of cases, *n* = 256) or MCI (23%, *n* = 135), and 33.4% (*n* = 196) were diagnosed with dementia or neurocognitive disorder owing to another aetiology. Applying all lecanemab eligibility criteria, 11 (4.3%) patients with dementia and two (1.5%) patients with MCI would have been eligible for treatment with this compound, whereas 13 dementia (5.1%) and 14 (10.4%) MCI patients met clinical inclusion criteria, but had no available amyloid status.

**Conclusions:**

Even in a memory clinic with a good infrastructure and sufficient facilities for dementia diagnostics, most patients do not meet the eligibility criteria for treatment with lecanemab.

Since the approval of donepezil in 1997, pharmacological treatment options for dementia owing to Alzheimer's disease are mainly limited to symptomatic therapy. In 2023, two monoclonal anti-amyloid antibodies were approved for use in the early clinical stages of Alzheimer's disease, but their feasibility in clinical practice remains unclear. In June 2021, the US Food and Drug Administration (FDA) granted accelerated approval for the first monoclonal anti-amyloid antibody,^1^ aducanumab, for the treatment of patients with mild cognitive impairment (MCI) or mild dementia owing to Alzheimer's disease. However, clinical efficacy has been questioned and it is considered controversial.^2^

In January 2023, lecanemab, another humanized immunoglobulin G1 monoclonal antibody, was approved by the FDA to treat patients in early clinical stages of Alzheimer's disease. Lecanemab is the first disease-modifying therapy, which significantly reduces amyloid burden and shows a statistically significant clinical benefit within 18 months. However, the clinical benefit is limited to a smaller decline in the Clinical Dementia Rating–Sum of Boxes score (CDR-SB) compared with placebo, but no improvement in cognition.^3^ Disease-modifying therapies for Alzheimer's disease are currently not available in Europe, but lecanemab is expected to be approved in 2024. In addition, donanemab, another monoclonal anti-amyloid antibody, is another compound showing positive biological and clinical results in a phase 3 clinical trial in patients with MCI and mild Alzheimer's disease dementia.^4^

## Disease-modifying therapies for Alzheimer's disease

Inclusion and numerous exclusion criteria of the clinical trials that led to FDA approval of lecanemab and aducanumab raised the question, how many patients are eligible for treatment with these agents in clinical routine? The Clarity AD phase 3 lecanemab trial reported that almost 60% of screenings failed because inclusion criteria were not met or exclusion criteria were present.^3^ There is reason for concern that an extensive screening of people for anti-amyloid therapy eligibility could reduce resources for those with other forms of dementia. In addition, the long-term benefits and possible negative effects of anti-amyloid therapies used to date are uncertain. For instance, the open-label extension lecanemab trial reported three deaths from amyloid-related abnormalities such as haemorrhages or encephalitis,^5^ and a systematic review and meta-analysis of magnetic resonance imaging (MRI) studies found that anti-amyloid therapy accelerated brain atrophy by approximately 39% compared with placebo.^6^ In addition, the very small slowing of cognitive decline observed with lecanemab therapy is sometimes questioned in terms of clinical meaningfulness.^7,8^

The introduction of disease-modifying therapies for Alzheimer's disease will require major changes in dementia care in clinical practice, and a detailed evaluation of real-world data and diagnostic standards for Alzheimer's disease is needed to guide patient selection and management strategies. Currently, most memory clinics do not have the necessary infrastructure for the safe administration of anti-amyloid antibodies. In addition to neuroradiologists for the sensitive detection of amyloid-related imaging abnormalities, specialists are needed to interpret amyloid positron emission tomography (PET) scans or cerebrospinal fluid analyses. Further, clinicians and neuropsychologists are needed to diagnose MCI owing to Alzheimer's disease or early Alzheimer's disease, as well as infusion centres that can handle a theoretically large number of patients.

## The role of memory clinics

It should be kept in mind that memory clinics not only provide diagnostic clarification of Alzheimer's disease, but also care for people with cognitive disorders of other aetiologies, which should not be neglected. In addition, clinical trials typically use strict eligibility criteria to achieve a homogeneous study population, reduce clinical and biological heterogeneity, and reduce safety and tolerability concerns. Therefore, comparability to everyday clinical populations is very low and needs to be captured in ‘real-world’ population-based analyses.

The current retrospective study of a real-world population of a psychiatric memory clinic provides information on how many patients would have been eligible for anti-amyloid treatment in routine clinical practice during a 19-month period. The results of this analysis show the difficulties in determining the indication for anti-amyloid treatment and the most common contraindications that preclude treatment with anti-amyloid antibodies.

## Method

### Study design

This was a retrospective, observational study to assess eligibility for lecanemab treatment in a psychiatric memory clinic population between 1 January 2022 and 31 July 2023. Data were collected from patients who had visited the memory clinic (Department of Psychiatry, Psychotherapy, Psychosomatics and Medical Psychology) at the Medical University of Innsbruck as part of routine clinical practice. The memory clinic at Innsbruck is one of three specialised out-patient clinics for the evaluation and treatment of patients with dementia and other neurocognitive disorders in Tyrol, Austria. Detailed biomarker diagnostics for amyloid and tau markers together with high-resolution imaging with 3-Tesla MRI are only available in these specialised memory clinics.

All patients completed a neuropsychological assessment and a clinical interview. Information on somatic comorbidities, the apolipoprotein (APO) ɛ genotype, as well as currently prescribed psychotropic and somatic medication, was obtained from medical records. Patients underwent cerebral imaging (MRI or computed tomography (CT)) and, if applicable, an amyloid PET scan or a fluorodeoxyglucose (FDG) PET scan. Inclusion criteria for eligibility assessment for treatment with lecanemab comprised a diagnosis of MCI as proposed by Petersen et al,^9^ or, if biomarkers were available, intermediate likelihood of MCI owing to Alzheimer's disease as proposed by Albert et al^10^ and probable or possible dementia owing to Alzheimer's disease as proposed by McKhann et al.^11^ MCI criteria^9^ included (a) cognitive complaints over the previous 6 months, reported by the patient or an informant; (b) impaired cognitive performance in one or more cognitive domains on the neuropsychological assessment of >1.5 s.d., corrected for age and education; (c) mild or no deficits in functional abilities and (d) diagnostic criteria of dementia not met. The following biomarker criteria (if available) were applied in addition to criteria 1–4 for diagnosing intermediate likelihood of MCI owing to Alzheimer's disease:^10^ (a) positive marker for neuronal injury (temporal cortical atrophy or temporal glucose hypometabolism in FDG-PET imaging) and (b) positive amyloid PET scan plus a Clinical Dementia Rating Scale (CDR)^12^ score of 0.5. Criteria for probable or possible dementia owing to Alzheimer's disease^11^ included (a) presence of memory impairment and disturbance of other cognitive function domains on the neuropsychological assessment of >2 s.d., corrected for age and education; (b) significant disruption of everyday life activities; (c) no disturbances in consciousness; (d) one or more emotional symptoms (e.g. emotional lability, irritability, apathy or disturbed social behaviour); (e) cognitive complaints over the previous 6 months reported by the patient and/or an informant and (f) positive marker of neuronal injury (atrophy of temporal lobe and/or medial parietal cortex) plus a CDR score ≥1. Cognitive impairment owing to other aetiologies or causes were diagnosed according to ICD-10 criteria.

The authors assert that all procedures contributing to this work comply with the ethical standards of the relevant national and institutional committees on human experimentation and with the Helsinki Declaration of 1975, as revised in 2013. All procedures involving human patients were approved by the Ethics Committee of the Medical University of Innsbruck, Austria (approval number 1046/2018). Because of the retrospective study design, patients were exempted from signing a consent form.

### Neuropsychological assessment

All participants completed a neuropsychological test battery including subtests of the Consortium to Establish a Registry for Alzheimer's Disease battery.^13^ The test battery included tests to assess verbal memory and recognition (word list learning, word list delayed recall and word list recognition), constructional praxis (figure drawing), figural memory (delayed recall), confrontational object naming (Boston Naming Test – short version), verbal fluency (animals/min, s-words/min) and cognitive flexibility (Trail Making Test A and B), as well as the Mini-Mental State Examination (MMSE).^14^ Age- and education-corrected *z*-scores were calculated from these measures.

### Questionnaires and scales

The Neuropsychiatric Inventory^15^ was used to assess the frequency (range: 0–4 points), severity (1–3 points) and emerging caregiver burden (0–5 points) of 12 behavioural and psychological symptoms of dementia.

Using the CDR, forgetfulness, difficulties in orientation, judgement and problem-solving, community affairs, home and hobbies, and care were evaluated by interviewing a caregiver or informant. An algorithm results in CDR scores ranging from 0 to 3 (0, normal cognition; 0.5, mild impairment; 1, mild; 2, moderate; 3, severe dementia).

Depressive symptoms were assessed with the 15-item version of the Geriatric Depression Scale.^16^ The questions were answered with ‘yes’ or ‘no’. The cumulative score is rated on a scoring grid. The grid sets a range of 0–5 as ‘not depressed’, 6–10 as ‘mildly depressed’ and 11–15 as ‘severely depressed’.

### Assessment of mental and somatic exclusion criteria for treatment with lecanemab

Electronic medical records were reviewed for current or past mental illness (e.g. affective disorder, substance misuse, psychosis, behavioural and psychological symptoms of dementia); somatic diseases, including cardiopulmonary (e.g. coronary artery disease, recurrent thrombosis, cardiac rhythm abnormalities), immunological (e.g. rheumatoid arthritis, Crohn´s disease, lupus erythematosus), central nervous system (e.g. epilepsy, hydrocephalus, history of intracerebral bleeding, stroke or transient ischemic attacks), renal and malignant neoplasms; and specific pharmacological treatment (e.g. anticoagulants, immunosuppressants, immunoglobulins, psychotropic drugs). Somatic disease, mental illness or pharmacological treatment were defined as contraindications to treatment with lecanemab according to the appropriate use recommendations.^17^ A mental or somatic condition was defined as unstable if the individual's condition had changed frequently and/or rapidly, had required constant monitoring and/or frequent adjustment of treatment regimens, and/or resulted in an emergency department visit or hospital admission within the past 12 months, based on current medical records.

### MRI acquisition

MRI data acquisition (Siemens Skyra/Verio, 3-Tesla scanner) used a predefined standardised protocol with a high-resolution T1-weighted three-dimensional MPRAGE sequence in 1 mm isotropic resolution coverage (repetition time: 1900 ms, echo time: 2.19 ms, inversion time: 900 ms, flip angle: 9°, isotropic resolution: 1 mm), an axial T2-weighted fluid attenuated inversion recovery (FLAIR) sequence (repetition time: 7230 ms, echo time: 97 ms, flip angle: 150°) and a diffusion tensor imaging sequence (repetition time: 7500 ms, echo time: 95 ms, 20 directions, field of view: 1610×1610 mm, flip angle: 90°, bandwidth: 1502 Hz/pixel, slices: 45). The susceptibility weighted imaging sequence was done using the following parameters: echo time: 28 ms, repetition time: 20 ms, flip angle: 15°, bandwidth: 120 Hz/pixel, slice thickness: 2.4 mm, slices per slab: 64.

White matter hyperintensities were rated visually by trained neuroradiologists using the Fazekas scale,^18^ on FLAIR or T2-weighted images. Medial temporal lobe atrophy was visually rated on coronal T1-weighted three-dimensional MPRAGE sequences.^19,20^ The presence of microbleeds/microhaemorrhages and superficial siderosis was assessed visually on FLAIR, T2-weighted and susceptibility weighted imaging sequences.

### Amyloid PET imaging

Amyloid imaging was performed with a GE Discovery MI 4 ring PET/CT scanner at 90 min after intravenous injection of 300 MBq ^18^F-florbetaben (Neuraceq®, Life Radiopharma GmbH, Berlin). The PET acquisition time was 10 min, and a low-dose CT was used for attenuation correction. PET data were co-registered with an MRI study, using the software provided by HERMES Medical Solutions. The nuclear medicine specialists were specially trained to evaluate the PET scans so that they could recognise whether significant amounts of amyloid plaques were present or not. Visual interpretation was made by comparing the activity in cortical grey matter with activity in adjacent cortical white matter. The lateral temporal, frontal, posterior cingulate, praecuneus and parietal lobes were systematically visually assessed and scored according to the regional cortical tracer uptake. A negative scan indicates a low or no density of cortical ß-amyloid plaques, and a positive scan indicates moderate to frequent density.

### Statistical methods

Patient characteristics were summarised with descriptive statistics (mean, s.d., interquartile range, count, percentage).

## Results

A total of 587 out-patients attended a scheduled appointment at the memory clinic for the assessment of memory complaints or as part of a routine control visit between 1 January 2022 and 31 July 2023.

Of all out-patients who presented to the clinic during the assessment period of 19 months, 391 (66.6%) met the diagnostic criteria for MCI or Alzheimer's type dementia and were selected as study population for eligibility analysis. Of the remaining 196 (33.4%) out-patients, 94 (16%) had dementia of other aetiology, 63 (2.2%) had mild to major cognitive impairment owing to other diseases and 55 (9.4%) had no significant cognitive impairment (Table 1).

**Table 1:** Diagnosis distribution and demographics of out-patients visiting the memory clinic between 1 January 2022 and 31 July 2023

Patient characteristics	Total out-patients
	*N* = 587
Demographics	Mean ± s.d. or *n* (%)
Age (years)	77.16 ± 9.67
	Range 36–98
MMSE (total score)	22.76 ± 5.89
	Range 2–30
Education (years)	10.67 ± 3.01
	Range 5–27
Amyloid status available (yes)	*n* (%)
	82 (13.9)
Diagnosis	*n* (%)
MCI	135 (23.9)
Amnestic MCI	96/135 (71.1)
Non-amnestic MCI	23/135 (17.0)
Multiple-domain MCI	16/135 (11.9)
Dementia	
Probable or possible dementia owing to Alzheimer´s disease	256 (43.6)
Dementia owing to Huntington's chorea	2 (0.3)
Parkinson's disease	13 (2.2)
Frontotemporal lobar degeneration	10 (1.7)
Vascular dementia	39 (6.6)
Dementia owing to another medical condition	30 (5.1)
Dementia owing to multiple aetiologies	10 (1.7)
Cognitive impairment primarily owing to	
Personality disorder	7(1.2)
Schizophrenia	8 (1.4)
Alcohol-related dementia	15 (2.6)
Major depression	29 (4.9)
Anxiety disorder	4 (0.7)
No objective cognitive impairment	55 (9.4)

MMSE, Mini-Mental State Examination; MCI, mild cognitive impairment.

### Clinical and demographic data

Clinical and demographic data of MCI and Alzheimer's disease dementia groups are summarised in Table 2. Out of the study population, 135 individuals (23%) were diagnosed with MCI and 256 (43.6%) with probable or possible Alzheimer's disease. All patients were White and residents of Austria, a country in Central Europe with a high-quality healthcare system and high educational and socioeconomic standards. A total of 64% of the patients were female, 6.1% were APOɛ4 homozygotes and 35% had an MMSE total score of <20 points. Hypertension and dyslipidaemia were the most prevalent cardiovascular comorbidities; 17.6% of patients were treated with non-vitamin K antagonist oral anticoagulants (acenocoumarol, warfarin, heparin, or cumarin) and 57% with one or more psychotropic medications (antidepressants, hypnotics or antipsychotics). A total of 75% of patients with dementia were treated with approved symptomatic medications for Alzheimer's disease (71% with acetylcholinesterase inhibitors, 4% with memantine). The amyloid status was available in merely 55 patients (14%).

**Table 2:** Demographic and clinic characteristic of all out-patients diagnosed with mild cognitive impairment and probable or possible Alzheimer's disease between 1 January 2022 and 31 July 2023

Variables	Mild cognitive impairment owing to Alzheimer's disease – intermediate likelihood	Probable or possible dementia owing to Alzheimer´s disease
	*N* = 135	*N* = 256
Demographics	Mean ± s.d. or *n* (%)	Mean ± s.d. or *n* (%)
Age (years)	74.56 ± 10.35	80.56 ± 6.85
50–90 years^a^	131 (97.3)	244 (95.3)
60–85 years^b^	109 (80.7)	197 (77.0)
65–85 years^c^	97 (71.9)	191 (74.6)
Male (%)	49 (36.3)	85 (33.2)
Female (%)	86 (63.7)	165 (64.5)
Education (years)	11.10 ± 2.99	10.37 ± 2.88
Amyloid status available (yes)	*n* (%)	*n* (%)
	16 (11.9)	39 (15.2)
Physical and cognitive scores	Mean ± s.d. or *n* (%)	Mean ± s.d. or *n* (%)
Apolipoprotein ε (% ε4-heterozygote)	32 (23.7)	69 (26.9)
(% ε4-homozygote)	6 (4.4)	18 (7.0)
Body mass index, kg/m^2^	26.15 ± 5.67	23.39 ± 3.41
<17 or >35^a^	3 (2.2)	18 (7.0)
MMSE (total)	26.43 ± 3.11	20.24 ± 5.81
22–30^a^	125 (92.6)	121 (47.3)
20–28^b,c^	99 (73.3)	155 (60.5)
GDS-15 (total)	4.92 ± 3.87	3.72 ± 3.01
>8^a^	23 (17.0)	15 (6.2)
CDR (total)	0.37 ± 0.23	1.68 ± 0.71
NPI (total)	3.45 ± 1.24	6.27 ± 3.43
Comorbidities	*n* (%)	*n* (%)
Diabetes	22 (16.3)	31 (12.1)
Hypertension	84 (62.2)	151 (59.0)
Dyslipidaemia	57 (42.2)	126 (49.2)
Coronary artery disease	29 (21.5)	35 (13.7)
History of seizures	5 (3.7)	3 (1.2)
Major cerebrovascular disease	17 (12.6)	50 (19.5)
Major depression	33 (24.4)	28 (10.9)
Substance misuse	7 (5.2)	18 (7.0)
Medication	*n* (%)	*n* (%)
Taking anticoagulants	22 (16.3)	47 (18.4)
Antithrombotic medication	48 (35.6)	62 (24.2)
Antihypertensives	85 (63.0)	144 (56.3)
Lipid-lowering medication	74 (54.8)	106 (41.4)
Oral antidiabetics	20 (14.8)	25 (9.8)
Insulin	10 (7.4)	8 (3.1)
Acetylcholinesterase inhibitor	1 (0.7)	180 (70.3)
Memantine	–	11 (4.3)
Antidepressants	75 (55.6)	106 (41.4)
Antipsychotics	32 (23.7)	47 (18.3)
Hypnotics	18 (13.3)	30 (11.7)

MMSE, Mini-Mental State Examination; GDS, Geriatric Depression Scale; CDR, Clinical Dementia Rating; NPI, Neuropsychiatry Inventory.

a. Inclusion criteria from the lecanemab Clarity AD trial (NCT03887455).

b. Inclusion criteria from the TRAILBLAZER-ALZ 2 trial (NCT04437511).

c. Inclusion criteria from the donanemab TRAILBLAZER-ALZ 3 trial (NCT05026866).

### Analysis of core eligibility criteria of phase 3 lecanemab and donanemab trials

Applying the age inclusion criteria of phase 3 lecanemab (Clarity AD)^3^ and donanemab (TRAILBLAZER-ALZ 2, TRAILBLAZER-ALZ 3) trials, 375 patients (96%) were in the age group for possible treatment with lecanemab and 288 (74%) for possible treatment with donanemab (Table 2). Based on the MMSE score inclusion criteria, 246 patients (62.9%) were eligible for lecanemab and 254 (65%) for donanemab therapy. In total, 239 (61%) out of 391 patients diagnosed with MCI owing to Alzheimer's disease or probable Alzheimer's disease met the core eligibility criteria in terms of age (50–90 years) and MMSE score^22–30^ for treatment with lecanemab. Applying the core eligibility criteria of the donanemab phase 3 TRAILBLAZER-ALZ 2 trial^4^ (age range 60–85 years, MMSE score 20–28) to the same group, the number of eligible patients narrowed to 201 (54%). Application of the ongoing phase 3 TRAILBLAZER-ALZ 3 trial criteria (age range 65–85 years, MMSE score 20–28) further reduced the number of eligible patients to 190 (49%).

### Analysis of eligibility of patients with MCI for lecanemab treatment

**Fig. 1:**
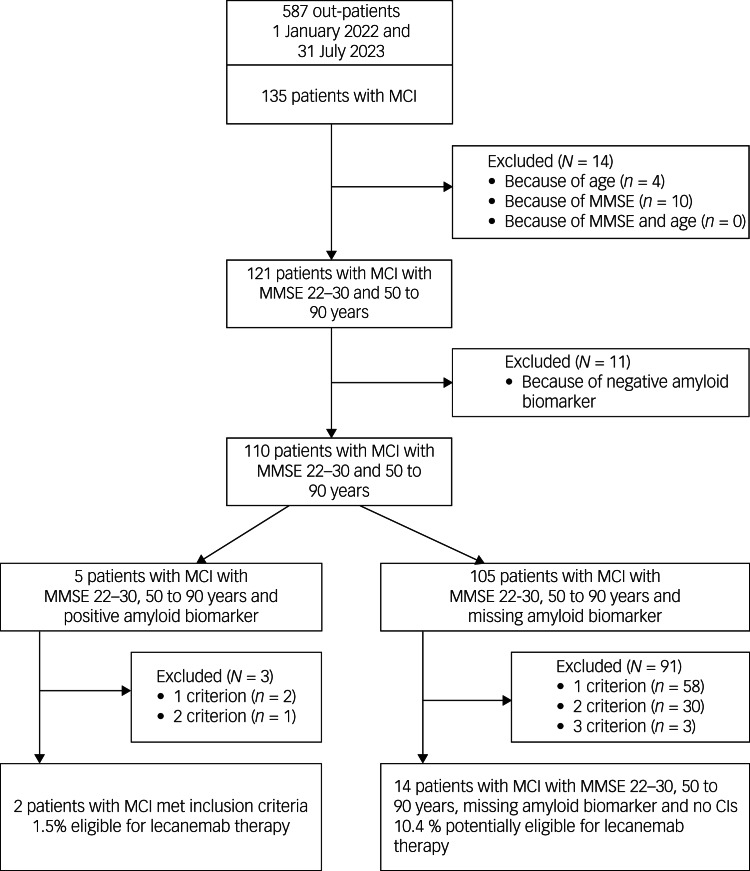
Study flow diagram and patient selection: mild cognitive impairment owing to Alzheimer's disease –intermediate likelihood. MCI, mild cognitive impairment; MMSE, Mini-Mental State Examination; CIs, contraindications.

Out of 135 patients with MCI, 14 did not meet the core inclusion criteria (age 50–90 years and MMSE score 22–30) for treatment with lecanemab (see Fig. 1, Table 3). Another 11 patients had to be excluded because of a negative amyloid biomarker status in amyloid PET. A positive amyloid PET scan was available in merely five out of the remaining 110 patients. Out of these, three had to be excluded because of exclusion criteria (APOɛ status, major depression or severe subcortical hyperintensities), resulting in a final number of two (1.5%) out of 135 patients with MCI meeting the inclusion criteria for treatment with lecanemab. Another 105 patients met the core inclusion criteria for treatment with lecanemab but had no evaluation of amyloid biomarker status. Applying further exclusion criteria for treatment with this compound in the latter group reduced the number of potentially eligible patients from 91 to 14, resulting in a total number of 16 (11.9%) potentially eligible patients with MCI, out of 135.

**Table 3:** Characteristics of participants with mild cognitive impairment with missing and positive amyloid biomarker status who met core eligibility criteria for lecanemab

Exclusion criteria based on recommendations for appropriate use for lecanemab and demographic data	Patients with MCI with MMSE 22–30, 50 to 90 years and positive amyloid biomarker	Patients with MCI with MMSE 22–30, 50 to 90 years and missing amyloid biomarker
	*N* = 5	*N* = 105
	Mean ± s.d. or *n* (%)	Mean ± s.d. or *n* (%)
No exclusion criteria	*n* = 2	*n* = 14
Age (years)	74.01 ± 7.07	73.71 ± 9.69
MMSE (total score)	26.50 ± 2.12	27.43 ± 1.51
With exclusion criteria based on recommendations for appropriate use for lecanemab	*n* = 3	*n* = 91
Age (years)	81.01 ± 7.01	75.33 ± 9.13
MMSE (total score)	26.33 ± 0.58	27.04 ± 2.03
MCI non-amnestic presentation	—	14 (13.3)
Body mass index <17 or >35 kg/m^2^	—	1(1)
Apolipoprotein ε4-homozygote	1 (33.3)	6 (5.7)
Unstable medical conditions	*n* (%)	*n* (%)
Coronary artery disease	—	2 (1.9)
Recurrent thrombosis	—	1(1)
Significant rhythm abnormalities	—	—
Severe renal disease with dialysis	—	—
Comorbidities/concomitant medication	*n* (%)	*n* (%)
Systemic treatment with immunosuppressant	—	2 (1.9)
Treatment with anticoagulants^a^	—	19 (18.1)
History and current seizures	—	1(1)
Malignant neoplasms	—	2 (1.9)
Severe mental illness		
Major depression	2 (66.6)	24 (22.9)
Substance misuse	—	5 (4.8)
Psychosis	—	8 (7.6)
Other comorbid mental illness	—	—
Cerebrovascular disease		
Microbleeds (>4)	—	3 (2.9)
Severe subcortical	1 (33.3)	32 (30.5)
Hyperintensities (Fazekas 3)		
Superficial siderosis	—	—
Lacunar infarcts (>2, past 1 year)	—	2 (1.9)
Transient ischemic attack (past 1 year)	—	2 (1.9)
History of inter-cerebral bleeding	—	
Hydrocephalus	—	1(1)
Multiple aneurysms	—	1(1)
Other exclusions	*n* (%)	*n* (%)
No care or family partner	—	2 (1.9)
No MRI available or possible	—	10 (9.5)

MCI, mild cognitive impairment; MMSE, Mini-Mental State Examination; MRI, magnetic resonance imaging.

a. Platelet aggregation inhibitors not included.

Detailed information on exclusion criteria found in patients with MCI is given in Table 3. The most prevalent was the presence of severe subcortical hyperintensities (30%), followed by major depression (23%) and treatment with anticoagulants (18%). In approximately 10% of patients with MCI with missing amyloid status, MRI was not available or possible, for example, because of a peacemaker or anxiety.

### Analysis of eligibility of patients with probable Alzheimer's disease for lecanemab treatment

**Fig. 2:**
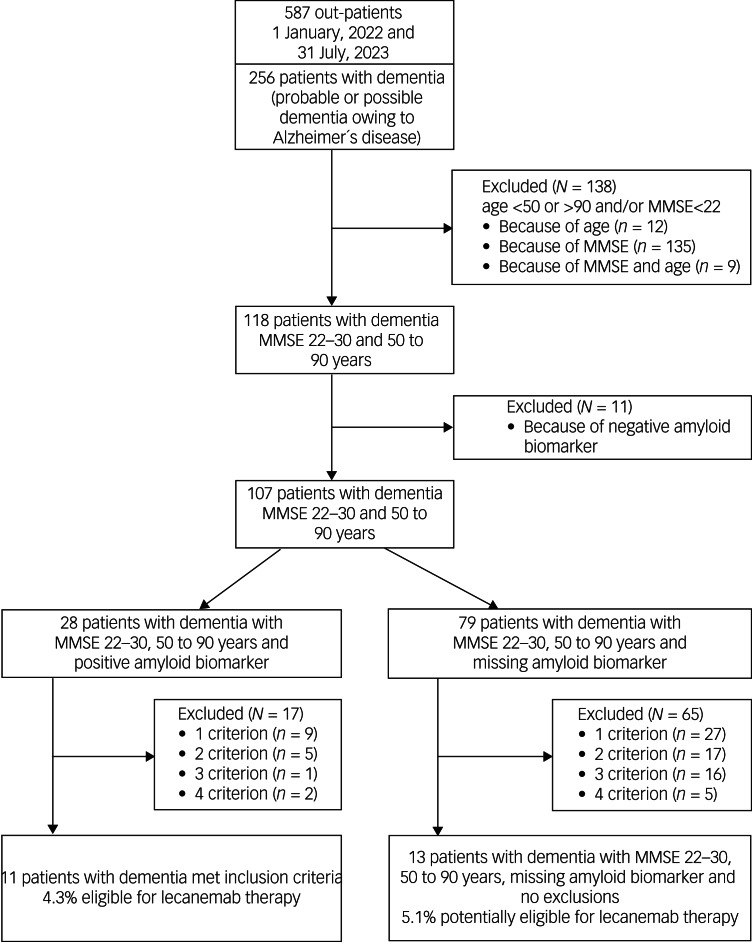
Study flow diagram and patient selection: probable or possible dementia owing to Alzheimer´s disease. MMSE, Mini-Mental State Examination.

A total of 138 (54%) out of 256 patients with probable Alzheimer's disease did not meet the core inclusion criteria (age 50–90 years and MMSE score 22–30) for treatment with lecanemab (see Fig. 2, Table 4). Another 11 patients had to be excluded because of a negative amyloid biomarker status in amyloid PET. Of the remaining 107 patients with dementia, 28 (26%) had an amyloid-positive PET scan, whereas the amyloid status of 79 patients (74%) was unknown. Of the 28 amyloid-positive patients, 17 had to be excluded because they fulfilled one or more of the four exclusion criteria. Finally, 11 (4.3%) out of 256 patients with dementia met the inclusion criteria for treatment with lecanemab.

**Table 4:** Characteristics of participants with dementia with missing and positive amyloid biomarker status meeting core eligibility criteria for lecanemab

Exclusion criteria based on recommendations for appropriate use for lecanemab and demographic data	Patients with dementia with MMSE 22–30, 50 to 90 years and positive amyloid biomarker *N* = 28	Patients with dementia with MMSE 22–30, 50 to 90 years, missing amyloid biomarker *N* = 79
	Mean ± s.d. or *n* (%)	Mean ± s.d. or *n* (%)
No exclusion criteria	*n* = 11	*n* = 13
Age (years)	76.73 ± 7.38	80.77 ± 4.04
MMSE (total score)	24.91 ± 2.12	24.46 ± 1.56
With exclusion criteria based on recommendations for appropriate use for lecanemab	*n* = 17	*n* = 65
Age (years)	77.01 ± 8.59	81.07 ± 4.93
MMSE (total score)	24.94 ± 2.36	24.26 ± 1.81
Body mass index <17 or >35 kg/m^2^	–	2 (2.5)
Apolipoprotein ε4-homozygote	4 (14.3)	7 (8.9)
Unstable medical conditions	*n* (%)	*n* (%)
Coronary artery disease	1 (3.6)	1(1.3)
Recurrent thrombosis	—	4 (5.1)
Significant rhythm abnormalities	—	1 (1.3)
Severe renal disease with dialysis	—	1 (1.3)
Comorbidities/concomitant medication	*n* (%)	*n* (%)
Systemic treatment with immunosuppressant	—	1 (1.3)
Immunologic disease	3 (10.7)	1 (1.3)
Treatment with anticoagulants^a^	3 (10.7)	16 (20.3)
History and current seizures	—	–
Malignant neoplasms	2 (7.1)	10 (12.7)
Severe mental illness		
Major depression	5 (17.9)	9 (11.4)
Substance misuse	—	6 (9.6)
Significant BPSD	—	4 (5.1)
Other comorbid mental illness	—	2 (2.5)
Cerebrovascular disease		
Microbleeds (>4)	1 (3.6)	3 (3.8)
Severe subcortical hyperintensities (Fazekas 3)	4 (14.3)	16 (20.3)
Superficial siderosis	—	4 (5.1)
Lacunar infarcts (>2, past 1 year)	5 (17.9)	6 (9.6)
Transient ischemic attack (past 1 year)	—	5 (6.3)
History of inter-cerebral bleeding	—	4 (5.1)
Hydrocephalus	—	3 (3.8)
Multiple aneurysms	—	2 (2.5)
Other exclusions	*n* (%)	*n* (%)
No care or family partner	1 (3.6)	2 (2.5)
No MRI available or possible	1 (3.6)	5 (6.3)

MMSE, Mini-Mental State Examination; BPSD, behavioural and psychological symptoms of dementia; MRI, magnetic resonance imaging.

a. Platelet aggregation inhibitors not included.

Analysis of the 79 dementia patients who met the core inclusion criteria and were missing amyloid biomarkers revealed 1–4 exclusion criteria in 65 cases (82%). Finally, only 13 patients from this group (5.1%) had no contraindication for treatment with lecanemab apart from the lack of amyloid evidence. This led to a total number of potentially eligible patients with probable Alzheimer's disease of 24 (9.4%) out of 256.

Detailed information on exclusion criteria found in patients with probable Alzheimer's disease is given in Table 4. As in patients with MCI, severe cortical and subcortical vascular pathology was most frequent (35%), followed by treatment with anticoagulants (20.3%) and malign neoplasms (12.7%).

### Change of eligibility for lecanemab over time

Of the 391 out-patients (135 with MCI, 256 with probable Alzheimer's disease), 77 came to one follow-up visit within the assessment period of 19 months. The two patients with MCI who met the inclusion criteria for treatment with lecanemab still met them at follow-up after 9–11 months. Of the 11 patients with dementia who met the inclusion criteria, four had a follow-up visit (follow-up time range: 9–13 months); two of them no longer met the inclusion criteria.

## Discussion

This study applied the eligibility criteria for treatment with lecanemab (phase 3 clinical trials and recommendations for appropriate use) to a psychiatric memory clinic population over a 19-month period. Out of 587 out-patients who came for clarification of memory complaints or presented in the context of a routine control visit, merely 13 (2.2%) individuals (two with MCI owing to Alzheimer's disease, 11 with dementia owing to Alzheimer's disease) would have met eligibility criteria for treatment with lecanemab. In 27 patients (4.6%; MCI owing to Alzheimer's disease: *n* = 14, dementia owing to Alzheimer's disease: *n* = 13), the lack of information on amyloid biomarker status was the only exclusion criterion. Applying the core inclusion criteria (diagnosis, MMSE score, age range) of the recently published donanemab phase 3 clinical trial^4^ and the ongoing donanemab TRAILBLAZER-ALZ 3 trial, the number of patients who possibly met eligibility criteria for amyloid antibody treatment was even lower. It has to be noted, however, that our study design is not feasible to provide a true estimate of eligibility for antibody therapies in the general population, but rather is applicable for patients treated in a high-quality public health system.

The vast majority of patients with MCI did not meet the eligibility criteria for treatment with lecanemab because of severe subcortical hyperintensities, major depression or treatment with anticoagulants. Surprisingly, the lack of availability of data on the challenging and expensive amyloid status was not the main reason for not meeting eligibility criteria in patients with MCI. We hypothesise that this high frequency of mental and/or somatic exclusion criteria in patients with MCI may be related to the relatively high age of our real-world study population (74.6 years) compared with, for example, the Clarity AD trial study population (71.2 years). We believe that these findings emphasise the importance of a comprehensive dementia diagnosis in younger people with MCI and the need to expand accessible screening and detection of Alzheimer's disease biomarkers, e.g. in the blood. A completely different picture emerged in the group of patients with Alzheimer's disease dementia, with over 50% being ineligible for treatment with lecanemab based on core age and MMSE criteria. Approximately 80% of the patients who met the core eligibility criteria had one to four other contraindications. Severe cerebrovascular disease and treatment with oral anticoagulants were the most common in this group.

Our results are only partially comparable with previously published work on the suitability for the already approved antibody therapies with lecanemab or aducanumab. In contrast to the eligibility data of previous studies,^21–23^ we analysed a routine memory clinic population outside of ongoing cohort studies or predefined populations within a clinical trial. Consequently, our out-patient population had a lower rate of amyloid biomarker analyses (11–14%). A similar retrospective study in a population with comparable rates of biomarker evidence found even lower rates (1%) of eligibility for treatment with aducanumab^24^ than we did for lecanemab. Another population-based cohort study reported 19 patients (8%) out of 5255 community-dwelling participants being eligible for treatment with lecanemab,^21^ and analyses of 2 870 023 patients with Alzheimer's disease and related disorders from the Centers for Medicare & Medicaid Services (CMS) found at least one exclusion criterion for treatment with aducanumab in 92% of patients.^22^ A possible indication for treatment with lecanemab or donanemab was identified in 906 people per year (1.1% of cases) in the analysis of 82 386 anonymous patient data from two UK databases. A national extrapolation based on these data showed a possible indication for treatment in 30 200 people in the UK per year, out of a total population of around 67 million.^25^ We believe that extrapolation from study cohorts to the general population without detailed knowledge of regional differences (e.g. in terms of comprehensive biomarker diagnostics and patient characteristics) should be interpreted with great caution.

Other groups found eligibility rates of 2–22% for treatment with aducanumab in biomarker-positive patients with MCI and Alzheimer's disease.^26,27^ In contrast to our data, no data on patients with memory impairment owing to other aetiology were provided, and a detailed analysis of exclusion criteria was missing. In addition, the study populations included only patients who met predefined inclusion criteria or gave informed consent for a research database. These may significantly limit the generalisability of these data. Only one retrospective study by Togher et al,^23^ which analysed suitability for aducanumab treatment, reported an eligibility rate of 57%. However, this study exclusively referred to patients with a positive amyloid biomarker status in cerebrospinal fluid, which substantially limits comparability with real-world memory clinic populations.

We conclude that many more patients with Alzheimer's disease could benefit from the new anti-amyloid treatment if biomarker diagnostics were also available in routine clinical practice for all patients. For this reason, current scientific research confirms the urgent need for the rapid establishment of cost-effective and widely available blood-based Alzheimer's disease biomarkers.^28^

It is also important to note that all of the aforementioned studies on eligibility for anti-amyloid treatment used inclusion and exclusion criteria from the phase 3 clinical trials that differ from published recommendations for the use of these agents.^17,29^ Although the recommendations on appropriate use of lecanemab regarding the core inclusion criteria (age, MMSE, body mass index) can be expanded based on physician judgement, the exclusion criteria regarding MRI findings or concomitant diseases are narrower.^17^ In particular, the strict recommended MRI exclusion criteria (e.g. evidence of amyloid beta-related angiitis (ABRA), cerebral amyloid angiopathy-related inflammation (CAA-ri)) are hardly applicable in clinical practice. Amyloid beta-related angiitis or inflammation are rare and possibly fatal, as well as underestimated, and final diagnosis requires brain biopsy.^30^ Some criteria for diagnosing ABRA or CAA-ri using clinical and MRI data have been proposed,^31^ but are not yet validated or implemented in routine neuroradiological imaging assessment. This is relevant because the risk of serious adverse events associated with amyloid-related imaging abnormalities is mentioned in the prescribing information of lecanemab-irmb (LEQUEMBI) and by an FDA black box warning. Our data provide evidence that risk factors for amyloid-related imaging abnormalities such as cerebrovascular disease and anticoagulant treatment are very common in patients with MCI and Alzheimer's disease dementia. Therefore, validated and clear MRI criteria are needed before administering anti-amyloid treatment to patients outside of clinical trials, to ensure safety. For this reason, we strongly support the use of anticoagulant treatment as a contraindication to anti-amyloid treatment as stated in the recommendations,^17^ in contrast to the Clarity AD exclusion criteria,^3^ until such MRI criteria are available.

Taken together, the data from different study populations and real-world data show that only a very small proportion of patients meet the inclusion criteria for any approved anti-amyloid treatment. This raises the question of whether the necessary restructuring of memory clinics and the cost-intensive biomarker screening can withstand a critical cost–benefit assessment. There may also be a risk that the expansion of care for people who have no chance of receiving anti-amyloid treatment will be postponed. Last, but not least, the clinical benefit of treatment with lecanemab must generally be viewed critically, especially as it is very low. Over 18 months, lecanemab treatment resulted in a smaller decline of only 0.45 points on the CDR-SB score compared with placebo,^3^ which is comparable with the meta-analysis of donepezil treatment.^32^

Nevertheless, anti-amyloid treatment options must be recognised as an important step forward in the treatment of people with MCI or Alzheimer's disease; however, eligibility criteria for using them, the published appropriate use recommendations and the prescribing information should be critically evaluated and standardised for use in routine clinical practice.

## Data Availability

The data that support the findings of this study are available on request from the corresponding author, M.D.
